# *Bacteroides fragilis* toxin (BFT) detection and neutralization using phage display-derived synthetic hagfish VLRBs

**DOI:** 10.1371/journal.pone.0361228

**Published:** 2026-11-02

**Authors:** Abigail Joy D. Rodelas-Angelia, Mark Rickard N. Angelia, Young Rim Kim, Cheolung Yang, Hyeok Jang, Jihyun Mun, Baek San Chang, Seong Wook Shin, Kim D. Thompson, Sang Soo Kang, Tae Sung Jung

**Affiliations:** 1 Laboratory of Aquatic Animal Diseases, Institute of Animal Medicine, College of Veterinary Medicine, Gyeongsang National University, Jinju-si, Gyeongsangnam-do, Republic of Korea; 2 Institute of Chemistry, College of Arts and Sciences, University of the Philippines Los Baños, Laguna, Philippines; 3 Earwynbio Co., Ltd., Jinju-si, Gyeongsangnam-do, Republic of Korea; 4 Moredun Research Institute, Penicuik, Scotland, United Kingdom; 5 Department of Anatomy, Institute of Medical Sciences, College of Medicine, Gyeongsang National University, Jinju-si, Gyeongsangnam-do, Republic of Korea; Universidade Federal do Rio de Janeiro, BRAZIL

## Abstract

Enterotoxigenic *Bacteroides fragilis* (ETBF) weakens the intestinal epithelial barrier and promotes inflammatory signaling through secretion of *B. fragilis* toxin (BFT), a metalloprotease associated with chronic intestinal inflammation. By triggering the breakdown of the E-cadherin glycoprotein in cellular junctions, BFT compromises tissue integrity and affects pathways involved in tumor suppression and signal transduction. Despite growing evidence implicating BFT in colorectal carcinogenesis, its role remains ambiguous, in part due to limited availability of suitable tools for its detection and neutralization. Selective binding molecules targeting BFT are, therefore, critical for understanding the role of ETBF in these clinical pathologies. Here, we used phage display screening to develop hagfish VLRB-derived consensus scaffold, termed ccombodies, as alternative non-immunoglobulin molecules targeting BFT. Three candidate binders (B8-1, C2-6, and G8-5) were expressed and purified for characterization. Specificity analyses against a range of bacterial virulence factors confirmed the selective recognition of BFT. Binding affinity was determined by ELISA titration and biolayer interferometry (BLI) yielding apparent K_D_ values in the nanomolar range. The ccombodies detected BFT at low nanogram levels by Western blot and exhibited high specificity and sensitivity in toxin recognition. Furthermore, treatment with ccombody attenuated BFT-induced cytotoxicity in Caco-2 and HT-29 epithelial cells. These findings establish ccombodies as a novel class of BFT-targeting molecules with potential applications in selective toxin detection, passive immunoprophylaxis, and antivirulence intervention.

## Introduction

Virulence factors are molecular accessories that bacterial pathogens deploy from its arsenal of tools to establish infection with the host. In most cases, the expression of specific virulence determinants distinguishes pathogenic strains from otherwise commensal members of the same bacterial species [1,2]. *Bacteroides fragilis* is a typical example, where non-toxigenic (NTBF) strains are common and beneficial residents of the gut but the toxigenic form (ETBF) secretes a zinc-dependent metalloprotease that fundamentally alters the host-microbe interaction [3,4].

*Bacteroides fragilis* toxin (BFT), or fragilysin, disrupts the intestinal barrier [5,6] by cleaving the transmembrane protein E-cadherin [7], which is essential for maintaining epithelial adherens junctions [8]. E-cadherin is a component of a protein–actin network that maintains epithelial structure and proteolytic cleavage leads to increased cellular permeability and enhanced exposure to ions, metabolites, and other toxins, thereby promoting immune activation [9,10]. Increasing evidence suggests that BFT contributes to colorectal cancer through the induction of these pro-inflammatory signaling pathways positioning BFT, among other bacterial metabolites, as a potential molecular marker that could improve our understanding of BFT-associated oncogenesis [11].

Detection of ETBF is achieved by the molecular identification of the *bft* gene using PCR [12–15], enterotoxin detection in cell-based assays [5,16,17], or immunoassay using monoclonal antibodies [18,19]. These approaches often require labor-intensive pre-analysis steps, which limits speed and practicality for routine analysis. Consequently, there is a need to expand the repertoire of BFT-binding molecules to provide more accessible and scalable approaches for ETBF-directed diagnosis and intervention [20].

To address this, we have developed synthetic hagfish-derived, variable lymphocyte receptor (VLR)-based molecules, termed ccombodies, that selectively target BFT. Hagfish VLRs are single-chain, crescent-shaped, LRR-rich molecules which specifically bind antigens and are functionally similar to conventional antibodies [21]. Here, BFT-binding ccombodies were identified through phage display screening and subsequently characterized for specificity, affinity, and functional activity. The resulting ccombodies demonstrated strong and selective recognition of BFT and conferred protection against toxin-mediated cytotoxicity in epithelial cell models, highlighting their potential as alternative molecules for studying and neutralizing ETBF virulence factors.

## Materials and methods

### Cell and bacterial culture

Caco-2 cells, obtained from the Korea Cell Line Bank, Seoul Korea (KCLB 30038), were cultured in 1x DMEM + Glutamax^TM^ (Gibco Life Technologies, USA) supplemented with 10% fetal bovine serum (FBS, Gibco Life Technologies), 1x non-essential amino acid solution, and 1x antibiotics (100 U/mL penicillin, 100 µg/mL streptomycin, and 250 ng/mL amphotericin B). HT29 cells (KCLB 30037.1) were cultured in Roswell Park Memorial Institute (RPMI) 1640 medium (Gibco Life Technologies) supplemented with 10% FBS and 1x antibiotics. *E. coli* TG1 cells (Agilent Technologies, USA) used in phage infection were inoculated in 2x Yeast extract-Tryptone (2x YT) medium (Formedium, England). *E. coli* BL21 competent cells (New England Biolabs, Ipswich, MA) were grown in Luria-Bertani (LB) medium (Difco Laboratories, USA).

### Phage library design and preparation

A consensus-derived synthetic hagfish VLRB molecule was used as the platform for phage display screening [22]. The molecular scaffold comprised of eight LRR modules, each containing four amino acid randomization sites (XLXLXX), within the hypervariable regions. For this screening, a library comprising more than 10^10^ variants was generated through selective randomization of module 3. The VLR scaffold was ultimately expressed as the N-terminal fusion to the M13 pIII minor coat protein. Prior to biopanning, the concentration of the phage library was diluted in blocking buffer A containing 1% bovine serum albumin (BSA) in PBST (phosphate-buffered saline; 135 mM NaCl, 2.7 mM KCl, pH 7.4, 0.05% Tween-20) to approximately 10^12^ plaque-forming units (pfu).

### Antigen coating and biopanning

Recombinant BFT antigen (BFT-1) was purchased from MyBioSource, Inc., USA (Cat. No. MBS1160138) and used in screening toxin-binding ccombodies. Sequence comparison verified that the recombinant toxin corresponded to the BFT-1 isoform. Five micrograms (5 ug) of the antigen, dissolved in 1 mL carbonate-bicarbonate buffer (pH 9.0), were added to a Polystyene Maxibinding immunotube (SPL Life Sciences) and incubated overnight at 4^o^ C. Blocking was subsequently carried out by incubating the immunotube with blocking buffer A for 1 h. The first round of biopanning was then performed according to the method of Kim and Ho [23], with modifications. Briefly, 1 mL of the phage library (10^12^ pfu) was incubated for 1 h in the antigen-coated, pre-blocked immunotube. Bound phages were eluted and used to infect early-log phase *E. coli* TG1 cells. Phage-infected cells were then cultured on a 2× YT plate (1% glucose and 100 µg/mL ampicillin) for at least 16 h at 30^o^ C. Colonies grown overnight were resuspended in 2x YT media (1% glucose and 100 µg/mL ampicillin), cultured to mid-log phase, and infected with 100 µL of M13KO7 (Antibody Design Laboratories) helper phage. After 1 h incubation at 37^o^ C and 200 rpm, the culture media was replaced with 200 mL of 2⨯ YT (100 µg/mL ampicillin, 50 µg/mL kanamycin, and 0.5 mM IPTG), cells were further incubated for at least 16 h at 30^o^ C and 200 rpm. Finally, the output phage particles were precipitated using 50 mL of polyethylene glycol 8000 (20% w/v PEG, 2.5 M NaCl) and pelleted via centrifugation. The phage particles were resuspended in 1⨯ PBS, and phage titer was adjusted to 10^12^ pfu for use in the next round of selection. A total of four rounds were performed for the enrichment of antigen-specific phage binders. After the final round of selection, 96 colonies were picked, grown to mid-log phase, infected with helper phage, and subsequently incubated overnight in a 96-well deep-well plate containing 200 µL/well of 2⨯ YT (100 µg/mL ampicillin and 50 µg/mL kanamycin). The resulting culture supernatants, containing ccombody-displaying phages, were then used in phage ELISA.

### Phage ELISA

Phage ELISA was performed according to the method described by Angelia et al. [24], with minor modifications. Recombinant BFT-1 protein (0.1 mL at 0.2 µg/mL) was coated onto a high-binding 96-well ELISA plate (Corning) and incubated overnight at 4 °C. An equivalent amount of BSA, used as a negative control, was coated onto a separate plate and processed under identical conditions. Both plates were then subjected to the following sequential steps: (a) blocking with 5% skim milk in 1× TBST (blocking buffer B; 10 mM Tris-HCl, 150 mM NaCl, 0.5% Tween 20); (b) incubation with culture supernatant (1:1 v/v, in blocking buffer B) obtained from biopanning; (c) incubation with HRP-conjugated M13 antibody (Sino Biological); (d) addition of developing buffer (42 mM 3,3′,5,5′-tetramethylbenzidine [TMB], 1% H_2_O_2_). Between each step, the plates were washed three times with 1× TBST, except after the final incubation step, where four washes were performed. Color development was terminated by the addition of 50 µL of 2 M H_2_SO_4_, and absorbance at 450 nm was measured using an xMark microplate reader (BIORAD).

### Cloning, expression, and purification of BFT-specific ccombodies

Following phage ELISA screening, twelve clones exhibiting the strongest BFT-binding signals were selected for sequence analysis. Clones sharing identical sequences were grouped, and one representative clone from each sequence group was selected for subsequent expression and purification. The target gene was subsequently amplified using appropriate primers and incorporated into a laboratory-developed shuttle vector via circular polymerase extension cloning (CPEC). The resulting plasmid was introduced into *E. coli* BL21(DE3) by electroporation for protein expression. Finally, antigen-specific ccombodies expressed in *E. coli* were purified using affinity purification with Ni-charged magnetic beads (PureCube INDIGO Ni-MagBeads), followed by fast protein liquid chromatography (FPLC) on Superdex TM 75 Increase 10/300GL column (ÄKTA). SDS-PAGE profiles of the fractions were analyzed using 12% acrylamide gels, and fractions containing the BFT-specific synthetic VLRBs were pooled for subsequent characterization.

### ELISA-based assessment of binding specificity

The binding specificity of the purified ccombodies was evaluated by ELISA using BFT (MyBioSource, MBS 1160138) as the target antigen, bovine serum albumin (BSA; Thermo Scientific) and bovine hyaluronidase (Sigma-Aldrich, H3506) as unrelated antigens, and a panel of recombinant bacterial virulence factors as heterologous antigens. The panel included *E. coli* cytolethal distending toxin subunit B (cdtB, Cusabio, CSB-EP671598ENL), *Staphylococcus aureus* α-hemolysin (Hla; Cusabio, CSB-EP639324FLFe1), *E. coli* β-lactamase (Sigma-Aldrich, L6170), *Cutibacterium acnes* lipase (Proteogenix, PPA2105), *Clostridium difficile* Toxin A (Native Antigen, CDA-TNL-50) and *C. difficile* Toxin B (Sigma, SML1153) representing virulence-associated proteins from other bacterial species, including gastrointestinal-associated pathogens. These proteins were selected to assess potential cross-reactivity with other bacterial virulence factors and were included as controls to assess nonspecific protein binding.

Briefly, the antigens were diluted in coating buffer (0.05 M carbonate-bicarbonate buffer, Sigma-Aldrich) and immobilized onto 96-well microtiter plates at a concentration of 100 ng/well by incubation at 4^o^C overnight. Wells were subsequently washed with washing buffer containing 0.02M Tris-HCl (pH 7.3), 0.05% (v/v) Tween 20, and 0.38 M NaCl and blocked with 5% (w/v) milk in washing buffer for 1 hr at room temperature. Following blocking, wells were washed and incubated with purified ccombodies at 200 ng/well for 1 hr. After washing, bound ccombodies were detected using an in-house anti-ccombody antibody 5F10. Wells were then incubated with 1:5000 (v/v) HRP-conjugated goat anti-mouse antibody (ThermoFisher Scientific) for 1 hr at room temperature. Wells were again washed thoroughly, and signal was developed using TMB. The reaction was terminated with 2M H_2_SO_4_, and absorbance was measured at 450 nm using an xMark BioRad microplate reader. Binding specificity was assessed by comparing the signal obtained with BFT to that observed for the heterologous antigens and BSA. Independent experiments were conducted in duplicate and repeated at least twice.

### Western blot analysis of BFT recognition

Serially diluted BFT antigen was first resolved by SDS-PAGE using a 12% acrylamide gel and subsequently electroblotted onto a nitrocellulose membrane using a Trans-Blot Turbo transfer system (BIORAD). The membrane was then blocked with 5% skim milk in 1× PBST (blocking buffer C), followed by incubation with purified BFT-specific ccombodies (diluted in blocking buffer C), mouse anti-ccombody antibody (5F10), and an HRP-conjugated goat anti-mouse antibody. Protein bands were visualized using SuperSignal West Pico PLUS Chemiluminescent substrate (ThermoFisher Scientific). The membrane was washed three times with 1× PBST between each step.

### ELISA-based EC_50_ dose-response assay

The half-maximal effective concentration (EC_50_) values for ccombody binding to BFT were determined by ELISA as described previously, with minor modifications. Each well was coated with 25 nM BFT and incubated for 1 h with serial two-fold dilutions of ccombodies (1.0 to 2.4 x 10^−7^ mg/mL). Bound ccombodies were detected with mouse anti-ccombody antibody (5F10, developed in-house). Color development was performed by adding TMB and H_2_O_2_. EC_50_ values were calculated by fitting the data to a four-parameter logistic (4PL) model using R version 4.5.2. Values are reported as mean of two independent measurements.

### Biolayer interferometry (BLI) analysis of binding kinetics

BLI was used to analyze the protein interaction kinetics between the ccombodies and the BFT antigen using an Octet R8 Protein Analysis System (Sartorius, Germany). Briefly, BFT was immobilized onto AR2G biosensors by EDC-NHS coupling in 10mM acetate buffer, pH 5, followed by a baseline measurement for 120 s in 1x Octet Kinetic Buffer. The antigen-loaded biosensors were then immersed in ccombody solutions at concentrations ranging from 9.375 to 500 nM for 240 s to monitor the association phase. Dissociation kinetics were then monitored for an additional 240 s in buffer-only solution. Experiments were independently performed thrice.

### Inhibition of BFT using ccombodies

Preliminary experiments were performed to determine the concentration of BFT-1 required to induce visible morphological changes in Caco-2 and HT-29 cells within 6 h of exposure. BFT-1 was serially diluted, and cellular morphology was monitored hourly by microscopy throughout the exposure period to identify the concentration producing the desired morphological response.

To determine whether the ccombodies inhibited BFT-mediated cellular damage, cells were washed once with serum-free medium and treated with the BFT-1 alone (10 nM for HT-29 and 50nM for Caco-2) or with BFT-1 pre-incubated with ccombody (1, 2, 4 mg/mL). Cells were incubated at 37^o^C for up to 12 h in a humidified atmosphere containing 5% CO_2_. Morphological changes were monitored by phase-contrast microscopy using a Nikon Eclipse Ti inverted microscope. The extent of cell rounding was scored at 5 h using a modified method based on Saidi and Sears [5], with the following scale: 1 + , 20–40% cell rounding; 2 + , > 40–60%; 3 + , > 60–80%; 4 + , > 80–100%. Representative images were captured at 5h.

In a separate experiment, Caco-2 cells were treated with BFT alone, BFT preincubated with ccombody, or ccombody alone for 1 h at 37^o^C and subsequently subjected to MTT assay using 3-(4,5-dimethylthiazol-2-yl)-2,5-diphenyltetrazolium bromide reagent (Vybrant MTT Cell Proliferation Assay Kit, Thermo Fisher Scientific) according to the manufacturer’s instructions. Before formazan solubilization, wells were examined by phase-contrast microscopy to assess cellular morphology. Following microscopic examination, dimethyl sulfoxide (DMSO) was added to dissolve the formazan crystals, and absorbance was measured at 540 nm. All experiments were performed independently at least twice, with three replicates per condition.

## Results

### Phage display library screening and selection of BFT-binding ccombodies

Phage display library screening was performed to identify ccombodies capable of binding BFT. Iterative enrichment yielded multiple phage clones displaying strong binding activity in phage ELISA. Binding signals of candidate clones were compared against a control plate containing the same concentration of BSA and twelve clones exhibiting strong BFT-binding signals were selected for sequencing (B8, C2, C6, C8, C10, D5, E8, F4, F7, G4, G8, and G9). Sequence analysis revealed three unique ccombody sequences among these clones. Clones C2 and C6 shared an identical sequence and were represented by C2, while G8, G9, and D5 shared an identical sequence and were represented by G8. The remaining clones (B8, C8, C10, E8, F4, F7, and G4) also shared an identical sequence and were represented by B8. Thus, the 12 phage-positive clones corresponded to three unique ccombody sequences, represented by B8, C2, and G8 (Fig 1A). These were cloned and expressed, and ELISA-positive expression clones were selected for affinity purification and FPLC, yielding B8-1, C2-6, and G8-5. SDS-PAGE analysis of FPLC fractions revealed bands consistent with the expected scaffold size of 32 kDa (Fig 1B-D). The purified ccombodies were then subjected to further binding characterization.

**Fig 1:**
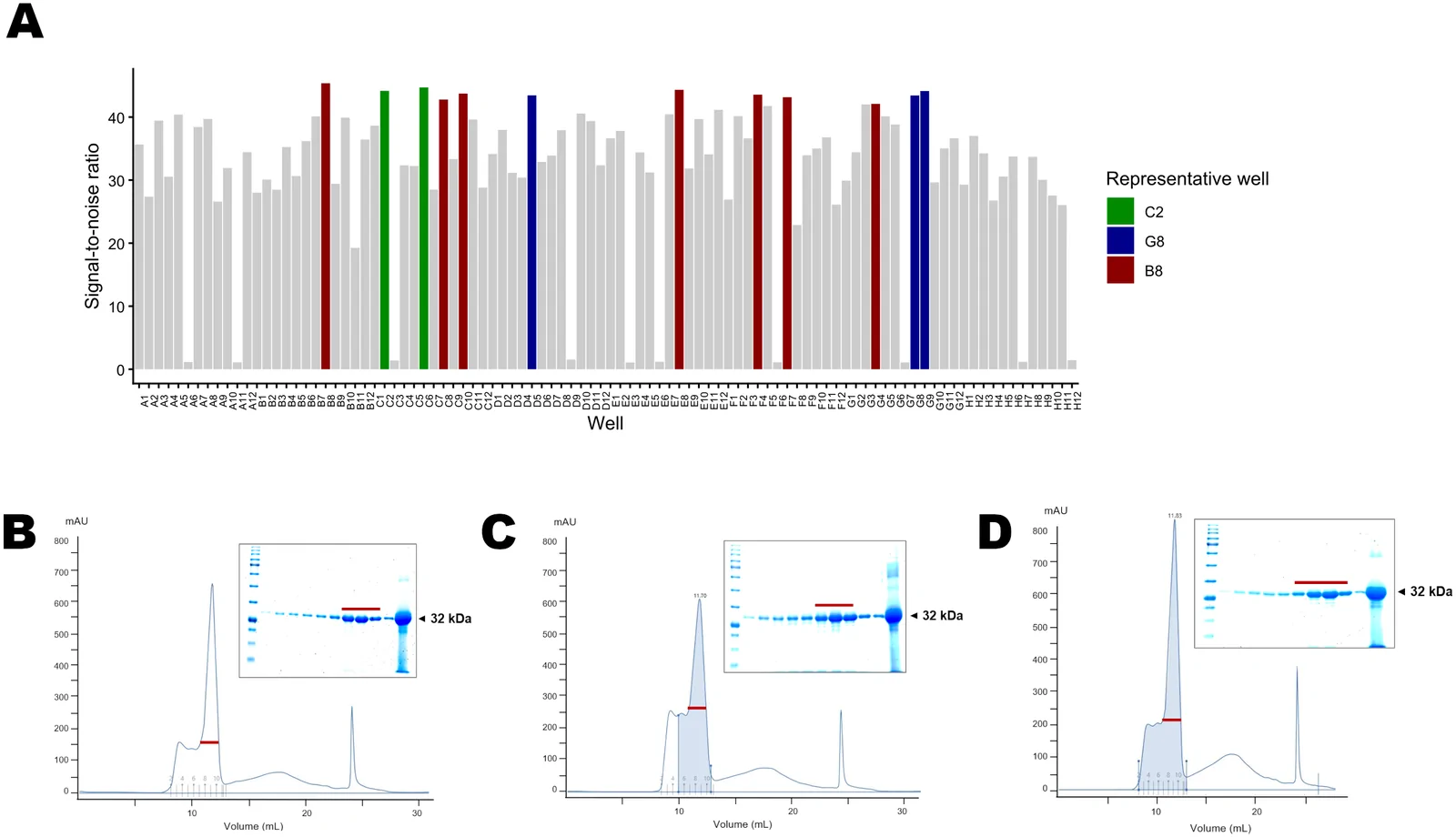
Identification and purification of BFT-binding ccombodies. (A) Phage ELISA screening of candidate binders. (B-D) Size exclusion chromatography (FPLC) profiles of expressed ccombodies (B: B8-1; C: C2-6; D: G8-5). (Inset) SDS-PAGE analysis of purification fractions (lane 1: marker; lanes 2-11: FPLC fractions; lane 12: magnetic bead eluate). The red bar indicates pooled fractions used for downstream analyses.

### Binding specificity and affinity

To evaluate the binding specificity of the ccombodies, ELISA was performed against the enterotoxin and a panel of unrelated proteins, bacterial virulence factors, and factors associated with gastrointestinal pathogens. All three candidates exhibited strong binding to BFT with minimal reactivity toward other proteins (Fig 2A), indicating selective recognition of the target antigen. Specificity was further validated by Western blot analysis using serially-diluted BFT. Each ccombody detected a single band corresponding to the expected molecular weight of ~60 kDa under denaturing conditions (Fig 2B). Among the variants, B8-1 demonstrated the highest sensitivity, detecting BFT at concentrations as low as 7.8 ng.

**Fig 2:**
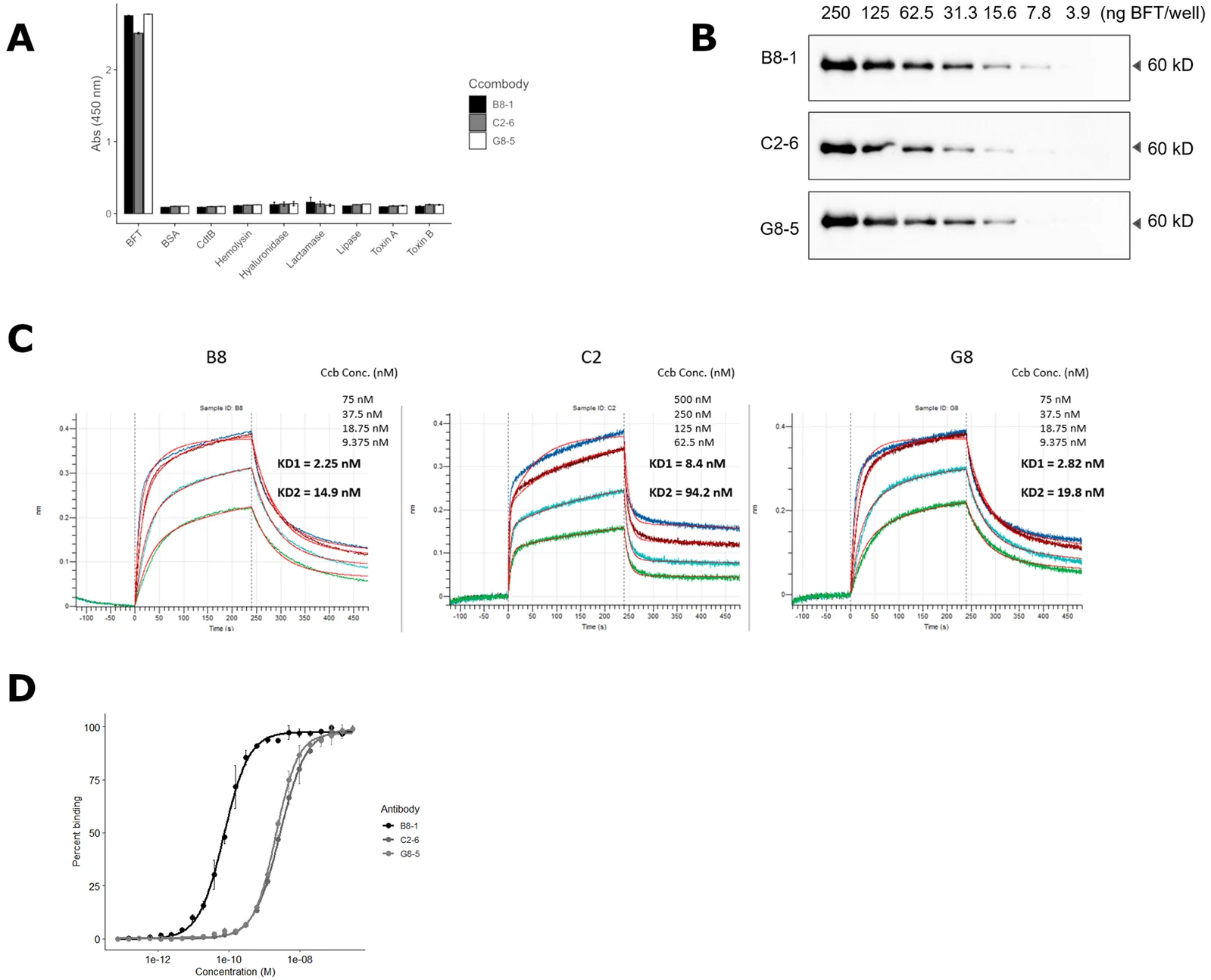
Specificity and binding affinity of ccombodies to BFT. (A) ELISA-based assessment of binding specificity against BFT and a panel of unrelated proteins (BSA and bovine hyaluronidase) and bacterial virulence- or pathogen-associated proteins (*E. coli* CdtB, *S. aureus* α-hemolysin, *E. coli* β-lactamase, *C. acnes* lipase, *C. difficile* toxins A and B.) (B) Western blot analysis of ccombody binding to serially diluted BFT. (C) BLI sensorgrams showing ccombody-BFT binding kinetics. (D) ELISA titration curves for EC_50_ determination.

### Binding kinetics

To quantify the binding interactions between BFT and the ccombodies, equilibrium dissociation constants (K_D_) were obtained by BLI, while EC_50_ values were determined by ELISA-based titration. Analysis of the association and dissociation phases revealed stable interaction across all candidates, with B8-1 and G8-5 displaying slower dissociation rates compared to C2-6 (Fig 2C). Sensorgram fitting was consistent with a heterogeneous ligand binding model, yielding apparent K_D_ values in the nanomolar range for all ccombodies (K_D1_: 2.25 ± 0.14, 8.42 ± 0.40, and 2.82 ± 0.09 nM; K_D2_: 15 ± 0.16, 94.3 ± 1.71, and 19.8 ± 0.26 nM for B8-1, C2-6, and G8-5, respectively; Table 1A).

**Table 1:** Functional and biophysical binding parameters of anti-BFT ccombodies.

A. BLI-derived binding constants of ccombodies (heterogeneous binding model).						
Ccombody	K_D_ (M)	K_D_ error	K_D2_ (M)	K_D2_ error	RSS	Full R^2^
B8-1	2.25 x 10^-9^	1.37 x 10^-10^	1.50 x 10^-8^	1.56 x 10^-10^	0.394	0.996
C2-6	8.42 x 10^-9^	3.95 x 10^-10^	9.43 x 10^-8^	1.71 x 10^-9^	0.4311	0.9951
G8-5	2.82 x 10^-9^	9.22 x 10^-11^	1.98 x 10^-8^	2.60 x 10^-10^	0.5155	0.995
B. ELISA-derived EC₅₀ values of ccombodies (four-parameter logistic model).						
Ccombody	EC_50_ (M)	SEM (M)				
B8-1	7.33 x 10^-11^	2.32 x 10^-12^				
C2-6	2.75 x 10^-9^	9.69 x 10^-11^				
G8-5	2.10 x 10^-9^	6.48 x 10^-11^				

Binding strength was further evaluated by ELISA-based titration using a four-parameter logistic model to estimate EC_50_ values. All three ccombodies exhibited concentration-dependent responses, with B8-1 showing the strongest apparent interaction and the lowest EC_50_ (73.3 ± 2.3 pM) among the variants (Fig 2D; Table 1B). Derived affinity parameters fall within the pico- to nanomolar range, consistent with strong binding interactions with the *B. fragilis* enterotoxin.

### Neutralization of cytotoxic activity

To determine whether the ccombodies bind to functionally important epitopes on BFT-1, toxin-induced cytotoxicity was evaluated using intestinal epithelial cell lines Caco-2 and HT-29. Exposure to BFT resulted in characteristic morphological changes, including cell rounding and cluster detachment (Fig 3, 4). Caco-2 cells exhibited lower sensitivity to the toxin, requiring either higher toxin concentrations or a longer incubation period to display visible levels of damage. Treatment with 50 nM BFT induced visible structural disruption in Caco-2 cells within 3–6 h, whereas 10 nM BFT was sufficient to induce cluster dissociation in HT-29 cells within the same incubation period.

**Fig 3:**
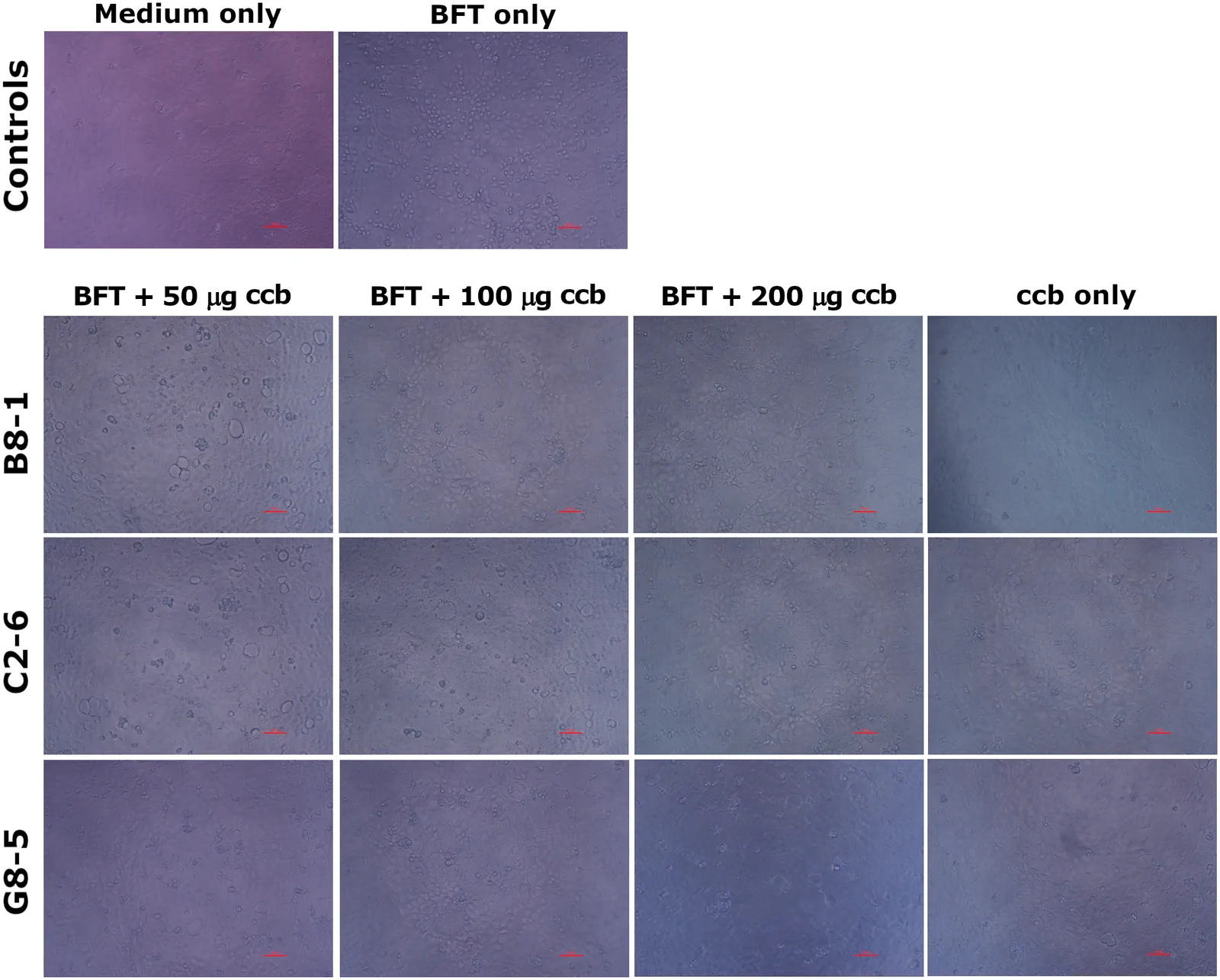
Ccombodies attenuate BFT-induced morphological damage in Caco-2 intestinal epithelial cells. Representative phase-contrast micrographs of Caco-2 cells following exposure to BFT in the presence or absence of ccombodies. Cells were treated with BFT alone, BFT pre-incubated with B8-1, C2-6, or G8-5 at 50, 100, or 200 µg, or ccombody alone. Media-only cells served as untreated controls. The corresponding ccombody-only conditions are shown in the rightmost column for each ccombody. Ccombody (ccb) refers to the synthetic VLRB-derived binding molecules. Images were captured by phase-contrast microscopy at 5 h after treatment. Scale bar, 100 µm.

**Fig 4:**
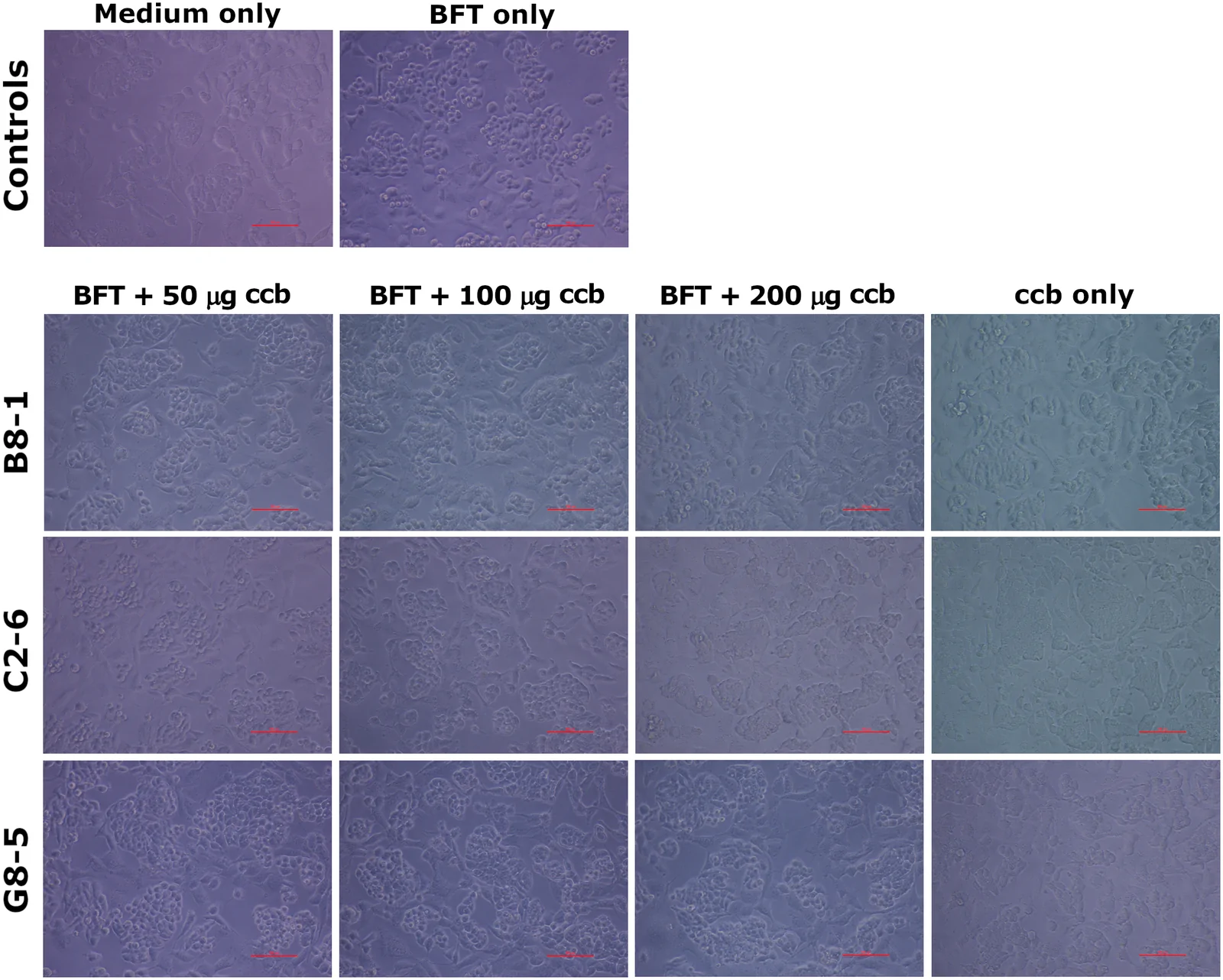
Ccombodies attenuate BFT-induced morphological damage in HT-29 intestinal epithelial cells. Representative phase-contrast micrographs of HT-29 cells following exposure to BFT in the presence or absence of ccombodies. Cells were treated with BFT alone, BFT pre-incubated with B8-1, C2-6, or G8-5 at 50, 100, or 200 µg, or ccombody alone. Media-only cells served as untreated controls. The corresponding ccombody-only conditions are shown in the rightmost column for each ccombody (ccb). Images were captured by phase-contrast microscopy at 5 h after treatment. Scale bar, 100 µm.

Pre-incubation of BFT with ccombodies (30 min – 1 h) attenuated these cytopathic effects in both cell lines, as evidenced by reduced cell rounding and preservation of cell-cell adhesion. This protective effect was concentration-dependent, with increasing ccombody concentrations (50, 100, 200 µg) resulting in a progressive reduction in BFT-induced morphological change (Table 2).

**Table 2:** Ccombody (ccb) inhibition of BFT-induced cell rounding in Caco-2 and HT-29 cells*.

Caco-2						
Anti-BFT ccombody	BFT only	BFT + 50 µg ccb	BFT + 100 µg ccb	BFT + 200 µg ccb	ccb only	Control
B8-1	++	–	–	–	–	–
C2-6	++	–	–	–	–	–
G8-5	++	–	–	–	–	–
**HT-29**						
Anti-BFT ccombody	BFT only	BFT + 50 µg ccb	BFT + 100 µg ccb	BFT + 200 µg ccb	ccb only	Control
B8-1	+++	++	++	–	–	–
C2-6	+++	++	+	–	–	–
G8-5	+++	++	++	+	–	

*Legend: (+)-sign estimates the extent of cell rounding per well (4 + , 80–100% cell rounding; 3 + , 60–80% cell rounding; 2 + , 40–60% cell rounding; 1 + 20–40% cell rounding)

To extend the observed protective effects of ccombodies on epithelial integrity and provide additional morphological context for the absorbance measurements in the MTT assay (S1 Fig), microscopic examination of MTT-treated wells before formazan solubilization was performed. Untreated cells formed a relatively continuous cellular layer, whereas BFT-treated cells exhibited pronounced disruption of the monolayer, including distinct acellular regions and increased accumulation of dark formazan precipitate in some remaining cells (Fig 5A, 5B). In contrast, cells treated with BFT pre-incubated with ccombody (Fig 5C) or ccombody alone (Fig 5D) retained greater cellular continuity and exhibited morphology more similar to that of untreated cells. These observations were consistent with attenuation of BFT-induced cellular disruption following ccombody treatment.

**Fig 5:**
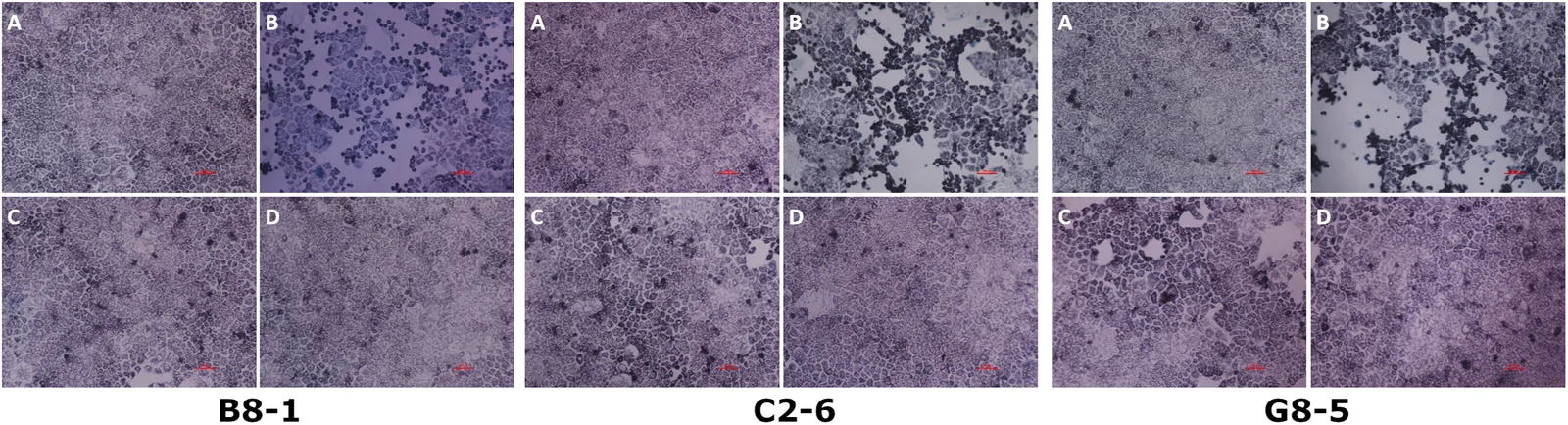
Microscopic examination of MTT-treated Caco-2 cells reveals attenuation of BFT-induced morphological disruption by ccombodies. (A) Cells in serum-free medium (untreated control). (B) Cells treated with BFT. (C) Cells treated with BFT pre-incubated with ccombody (BFT + 50 µg ccombody). (D) Cells treated with ccombodies only (200 µg). Micrographs were captured before formazan solubilization.

## Discussion

In this study, we report three novel hagfish VLRB-based molecules, termed ccombodies, that detect BFT from ETBF and attenuate its effects in epithelial cell models *in vitro*. The ccombodies were isolated using a phage display platform which shows strong potential for screening binders against bacterial virulence factors in addition to previously reported viral protein targets [22,25] and glycan moieties [24]. To our knowledge, this is the first report of a virulence factor-targeting, hagfish VLRB-based ccombody with functional properties that may be useful for detection, passive immunoprophylaxis, and antivirulence therapy.

BFT expression distinguishes pathogenic ETBF from commensal *B. fragilis* strains and represents an attractive and specific diagnostic marker. Detection strategies primarily rely on *bft* gene or BFT protein assays but these commonly used approaches possess notable limitations and require specialized laboratory infrastructure [20,26]. PCR-based *bft* gene detection methods are sensitive and may be used to classify BFT isotypes [12,27–29], but are also primer-dependent and may lead to inconsistent results [30]. BFT-induced morphological changes in cell culture detect functionally active toxins [5,16] and have been instrumental in elucidating the mechanisms of BFT activity [6,31–33] but may be subject to observation bias and can have limited sensitivity in complex clinical samples. Often, bacterial culture is required prior to gene or protein analysis and is, therefore, time-consuming and labor-intensive [14]. These constraints emphasize the need for alternative diagnostic options for routine use especially in physiologically relevant environments [19,20,34].

Monoclonal antibodies have also been developed for standardized detection and neutralization of BFT and have demonstrated efficacy in in vitro assays of BFT activity [18,19]. However, widespread application can be constrained by factors such as large molecular size, complex production requirements, limited tissue penetration, and stability [35]. Recently, alpaca-derived nanobodies have demonstrated that small antibody fragments can effectively detect BFT and, in some cases, bind catalytic epitopes resulting in the neutralization of the toxin’s biological activity [36].

LRR-containing modular scaffolds, including lamprey and hagfish VLRBs, have emerged as promising binding platforms that may be weighed as alternatives to conventional immunoglobulin-based molecules [37,38]. Lamprey-derived antibodies, in particular, have been successfully applied to target clinically relevant and technically challenging antigens and, in some cases, have displayed better performance relative to conventional Ig-based antibodies [39–42]. These advances highlight the potential of engineered high-affinity proteins, including VLR-based scaffolds and single-domain antibodies, as next- generation probes for translational applications.

In a similar context, our findings show that hagfish VLRB-derived, consensus-designed molecules can be engineered to selectively recognize BFT (Figs 1 and 2), representing an available non-immunoglobulin binding scaffold option against the *B. fragilis* enterotoxin. We obtained three unique ccombodies following a hagfish-based VLRB consensus sequence with randomized amino acids in module 2 which contains LRR3 and LRR4 in the hypervariable region of the scaffold. LRR cassettes in the concave surface of ccombodies which, similar to lamprey VLRBs, correspond to the antigen-recognizing region and specificity determinant of the molecule [43]. Randomizing the amino acids corresponding to this hypervariable region, XLXLXX, yields a diverse set of molecules capable of binding to various types of antigens. Combining this diversity with phage display serves as an efficient platform for generating synthetic ccombodies with high binding specificity and affinity towards a molecular target.

Across complementary protein interaction assays, the identified ccombodies exhibited consistent evidence of target recognition. In ELISA (Fig 2A), strong and selective binding of BFT was observed using a panel consisting functionally unrelated proteins, bacterial virulence factors, and gastrointestinal pathogen-related toxins. In Western blot analysis (Fig 2B), ccombodies demonstrated detection at low nanogram levels under denaturing conditions, broadly within the detection range reported for commercially available polyclonal antibodies against BFT. Manufacturer-provided Western blot demonstrated BFT detection over the tested range of 10–80 ng, whereas our ccombodies yielded detectable signals over the tested range of 7.8–250 ng under the conditions used in this study. Although direct quantitative comparison is limited because the ccombody and antibody data were not obtained in head-to-head experimental conditions. Furthermore, although polyclonal antibodies against BFT are commercially available, they are limited and mainly applied in ELISA- and Western blot-based detection methods and do not have publicly available information on their K_D_ values or demonstrated toxin-neutralizing activity. Thus, the development of alternative BFT-binding molecules remains relevant and the reported analytical performance of anti-BFT commercial reagents from Cusabio and Thermo Fisher Scientific provides a useful benchmark for BFT detection. Quantitative analyses further corroborated ELISA and Western blot findings, with ELISA-based EC_50_ measurements and BLI-derived K_D_ values indicating stable interactions with the toxin (Figs 2C and 2D, Table 1). These results highlight the potential of ccombodies as toxin-specific binders and support their application in the selective detection and quantification of BFT. Further investigation is warranted to assess their performance in diagnostic applications and in the analysis of clinical samples.

Notably, ccombody treatment attenuated BFT-induced cytotoxicity in intestinal epithelial models, as demonstrated by decreased cell rounding and aggregation in both Caco-2 (Fig 3) and HT-29 cells (Fig 4). These morphological changes are characteristic of BFT activity and arise from proteolytic cleavage of the extracellular domain of the transmembrane protein, E-cadherin, leading to disruption of adherens junctions and destabilization of the actin cytoskeleton through associated catenin complexes [7,44]. While the precise mechanism of inhibition remains to be defined, we hypothesize that ccombodies partially occlude the active site of BFT, thereby reducing its ability to cleave E-cadherin.

Consistent with previous reports [16], HT-29 cells exhibited greater sensitivity to BFT than Caco-2 cells. Although ccombody treatment conferred protective effects in both models, it effectively halted toxin-induced morphological changes in Caco-2 cells, whereas only a modest attenuation of toxin effects was observed in HT-29 cells. In addition, BFT-treated Caco-2 cells displayed surface protrusions resembling membrane blebbing, further supporting cytoskeletal disruption, as initially observed by Donelli et al. [31], which was mitigated in the presence of ccombodies. Microgram-level concentrations of ccombody used in the functional neutralization assay were selected based on previous experimental experience with ccombody-based assays in our laboratory. At 50 nM BFT, the ccombody concentrations tested, 50–200 µg, represented a substantial molar excess relative to the toxin, however, the present experiment was designed to establish functional neutralization under defined assay conditions rather than determine the minimum inhibitory concentration of the ccombody. For the conditions examined in HT29 cells, microgram-levels of the ccombody were required to attenuate BFT-mediated cytotoxic effects. One possible explanation is that the monovalent nature of recombinant ccombodies may result in lower apparent avidity than that of multivalent antibody-based reagents. However, native hagfish VLRBs have been reported to form oligomeric complexes [45] and whether the oligomeric state of the ccombodies contributes to their neutralization efficacy in functional assays remains to be experimentally determined. Concentration-dependent studies will therefore be necessary to define the lowest effective concentration for ccombody-mediated BFT neutralization.

Consequently, the functional activity of the ccombodies can be considered in relation to approaches used to evaluate previously reported BFT-targeting antibodies. The monoclonal antibody ICT11 was reported to detect BFT by ELISA and Western blot and to neutralize ETBF-induced cell rounding in HT-29/C1 cells using 50µg/mL BFT crude extract and 1:20 dilution of the mAb [18]. BFT neutralization was assessed based primarily on qualitative observation of toxin-induced morphological changes, similar to the method used in the present study. Mootien and Kaplan [19] subsequently generated 22 monoclonal antibodies recognizing isotypes BFT-1, BFT-2, or both, among which mAb 1D9 reduced BFT-1-induced cytotoxicity and E-cadherin release in HT-29/C1 cells. This study used both HT29/C1 cellular morphology evaluation (using 1µg/well BFT with 10 µg mAb) and ELISA-based E-cadherin measurements which provided complementary evidence of functional toxin inhibition. More recently, Guo et al. [36] reported a single-domain antibody, Nb2.43, that inhibited BFT-1-mediated E-cadherin cleavage (using 3µg/mL aBFT-1 with 100 µg/mL Nb). Neutralization was assessed by E-cadherin-based Western blot and immunofluorescence analyses. In the present study, all three ccombodies attenuated BFT-1-induced morphological changes in HT-29 and Caco-2 cells when treated with ccombody at 50, 100, and 200 µg/well, while exhibiting apparent K_D_ values in the nanomolar range by BLI. These findings provide a useful point of reference for comparison with previously reported BFT-targeting antibodies. However, direct quantitative comparison is limited because these studies were not performed under similar assay conditions. In particular, the morphological assay used in the present study provides evidence of protection against a BFT-induced cellular phenotype but does not establish whether the ccombodies directly inhibit BFT proteolytic activity or prevent E-cadherin cleavage. Nevertheless, the ability of three independently selected ccombodies to combine specific BFT recognition with attenuation of BFT-induced cellular effects demonstrates that antibody-like functional activity can be achieved using a non-immunoglobulin VLRB-derived scaffold. These findings support ccombodies as an alternative molecular format for BFT targeting, while further biochemical characterization and direct head-to-head studies under standardized conditions will be necessary to determine their relative functional performance. Furthermore, the continued discovery of BFT-targeting antibodies and other binding molecules further reflects sustained scientific interest in generating reagents for BFT recognition and functional targeting.

To further characterize the effects of BFT on Caco-2 cells and the effects of ccombody treatment, cellular responses were evaluated using an MTT assay. We hypothesized that BFT-induced cytotoxicity would reduce metabolic activity and consequently decrease formazan production. However, MTT absorbance measurements did not show the expected decrease following BFT exposure. We instead observed a marginal increase in absorbance compared to untreated cells (S1 Fig). This unexpected response indicates that MTT absorbance alone may not adequately reflect the extent of BFT-induced cellular damage under these experimental conditions and, therefore, should not be interpreted as a direct measure of cell viability in this context. The response may be associated with changes in cellular morphology, permeability, or MTT and formazan accumulation following disruption of epithelial integrity. This observation is also consistent with previous reports suggesting that BFT-induced cellular effects can involve structural and functional alterations that may precede or occur independently of immediate cell death [7,31,46]. Sanfilippo et al. [47] similarly reported minimal changes in mitochondrial dehydrogenase activity following BFT exposure in Caco-2 cells compared with T84 cells, suggesting that cellular responses to BFT may vary according to differences in cell-surface composition and architecture.

To provide morphological context for the unexpected MTT absorbance measurements, MTT-treated wells were examined microscopically before formazan solubilization. BFT exposure resulted in substantial disruption of the cellular monolayer, including occasional loss of cell–cell adhesion, altered cellular morphology, and distinct acellular regions. In contrast, cells treated with ccombody alone or BFT pre-incubated with the ccombodies retained greater cellular continuity and exhibited morphology more similar to that of untreated cells (Fig 5). These observations provide a morphological correlate of the cellular effects of BFT and a supporting interpretation of the MTT results in conjunction with the underlying cellular morphology. These quantitative and microscopic observations indicate that ccombody treatment was associated with preservation of cellular integrity following BFT exposure.

These findings indicate that the ccombodies not only recognize BFT-1 but also attenuate BFT-mediated cytoskeletal disruption. However, the molecular mechanism underlying this attenuation was not established in the present study. Since E-cadherin is a known substrate of BFT, through binding with claudin-4 [48], the observed protective effects may result from interference with BFT-mediated E-cadherin cleavage or other steps involved in toxin–cell interactions. However, this possibility remains to be experimentally determined. In addition, the specificity of the current ccombodies toward other BFT variants, including BFT-2 and BFT-3, remains unknown. Future studies should therefore investigate both the ability of these ccombodies to distinguish among BFT subtypes and the molecular basis of their protective activity against BFT-induced cellular damage. Such studies may further define the utility of ccombodies for BFT detection and functional targeting, particularly in light of the potential relevance of BFT variants as disease-associated biomarkers [49], while also providing mechanistic insight into ccombody-mediated attenuation of toxin activity.

Evidence of ccombodies as protease-targeting inhibitors strongly emphasizes their potential in anti-virulence strategies for the control of ETBF pathogenicity. Neutralizing virulence factors through monoclonal antibodies has received attention as an approach to mitigate infection while reducing selective pressures that drive antimicrobial resistance [50]. This strategy is particularly relevant for organisms such as *B. fragilis*, characterized by its capacity to acquire virulence-associated genes [51] and is increasingly recognized for its multidrug resistance [52]. Furthermore, the ccombody phage display platform utilized in this study provides a potentially invaluable framework for the discovery of anti-infective agents against key virulence determinants.

In summary, this study demonstrates the successful development of hagfish-derived VLRB-based ccombodies as novel, non-immunoglobulin binding molecules targeting *Bacteroides fragilis* toxin (BFT). Through a combination of biochemical and functional analyses, we show that these ccombodies exhibit selective and high-affinity binding to BFT and effectively mitigate toxin-induced epithelial disruption *in vitro*. By preserving epithelial integrity without directly targeting bacterial viability, ccombodies represent a promising antivirulence strategy with potential translational applications in both toxin detection and therapeutic intervention. Moreover, this work expands the utility of VLR-based scaffolds as adaptable platforms for targeting clinically relevant and technically challenging virulence factors.

## Supporting information

S1 Fig MTT assay of Caco-2 cells following BFT and ccombody treatment. Caco-2 cells were treated with 50nM BFT alone, BFT pre-incubated with ccombody at 50, 100, and 200 µg, or with 200 µg ccombody alone. Untreated cells were used as control. Experiments were performed independently at least twice, with three replicates per condition.
(TIFF)

S1 File Original uncropped blots and gels. Raw and uncropped images of SDS-PAGE gels and Western blots presented in Figs 1 and 2.
(PDF)
