# Supplementary material for: Bacteroides fragilis toxin (BFT) detection and neutralization using phage display-derived synthetic hagfish VLRBs
Source: PLoS One. 2026 Nov 2;21(11):e0361228. doi: 10.1371/journal.pone.0361228 (PMC13726912; doi:10.1371/journal.pone.0361228)
Supplement: Supplementary file 1 [file pone.0361228.s002.pdf]

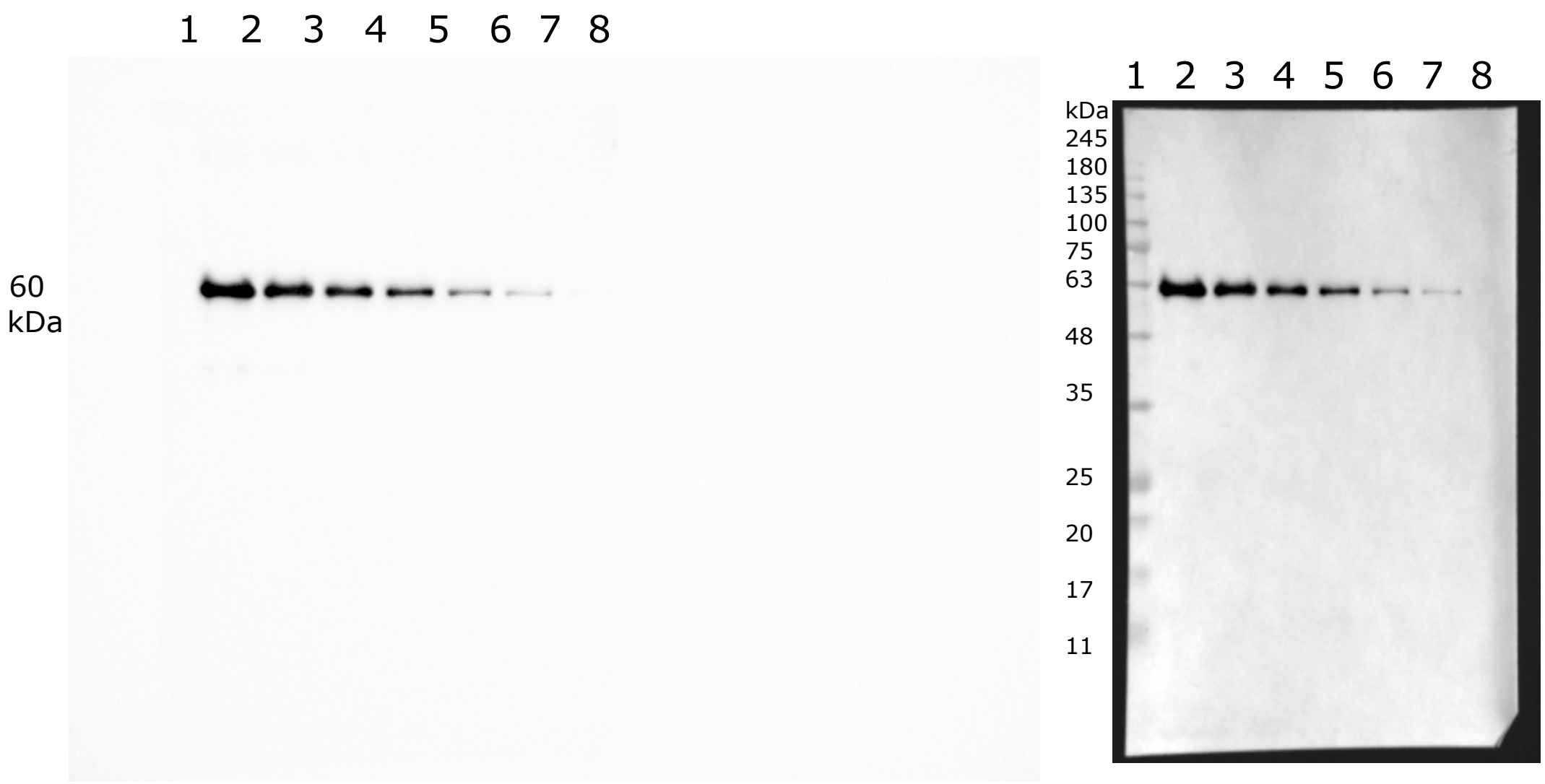

Raw image file for Fig 2B **(B8-1)**
Left blot: chemiluminescent image
Right: colorimetric merge

Samples
1 Molecular weight marker
2 250 ng BFT
3 125 ng BFT
4 62.5 ng BFT
5 31.25 ng BFT
6 15.625 ng BFT
7 7.8125 ng BFT
8 3.90625 ng BFT

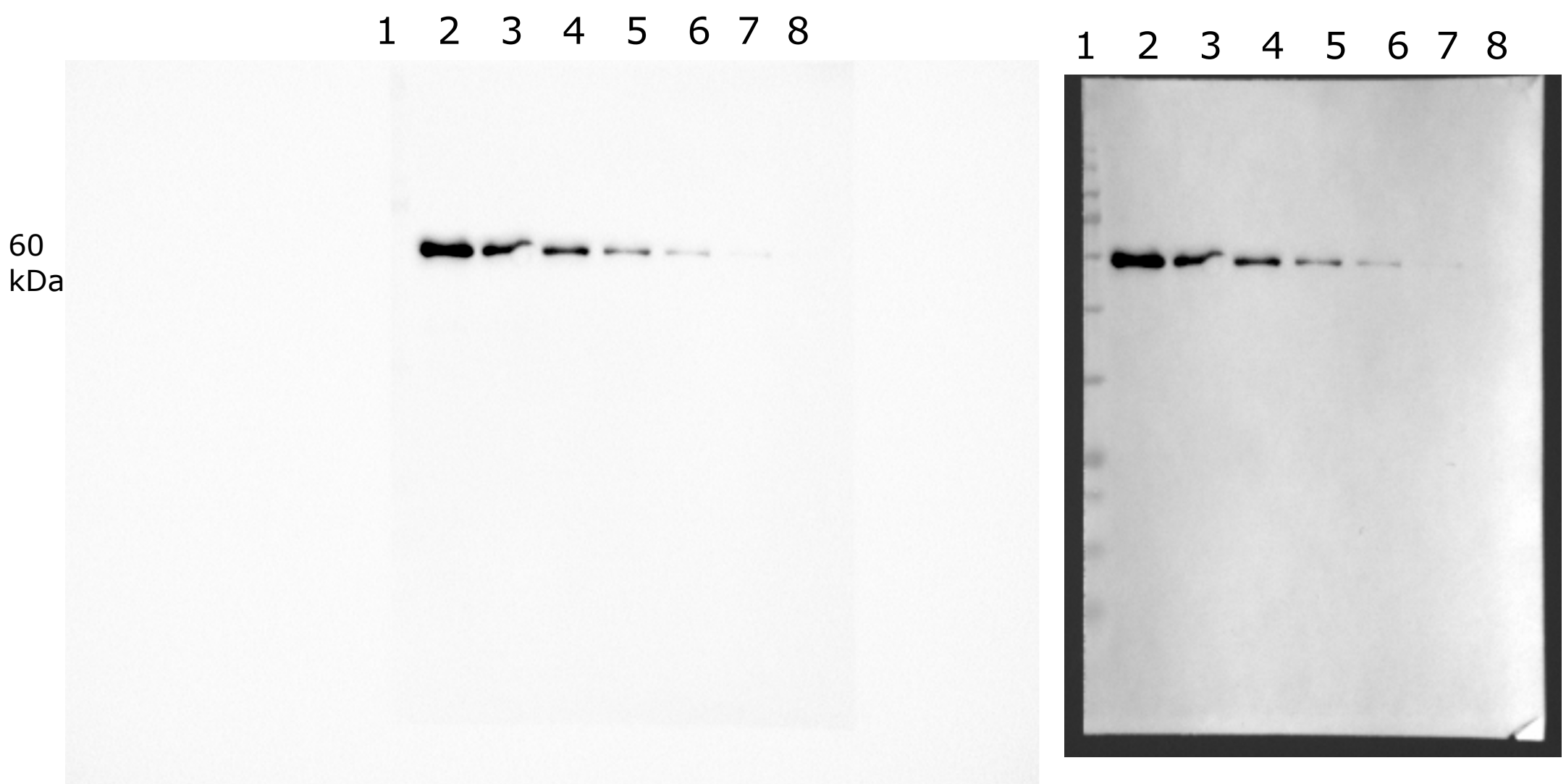

Raw image file for Fig 2B **(C2-6)**
Left blot: chemiluminescent image
Right: colorimetric merge

Samples
1 Molecular weight marker
2 250 ng BFT
3 125 ng BFT
4 62.5 ng BFT
5 31.25 ng BFT
6 15.625 ng BFT
7 7.8125 ng BFT
8 3.90625 ng BFT

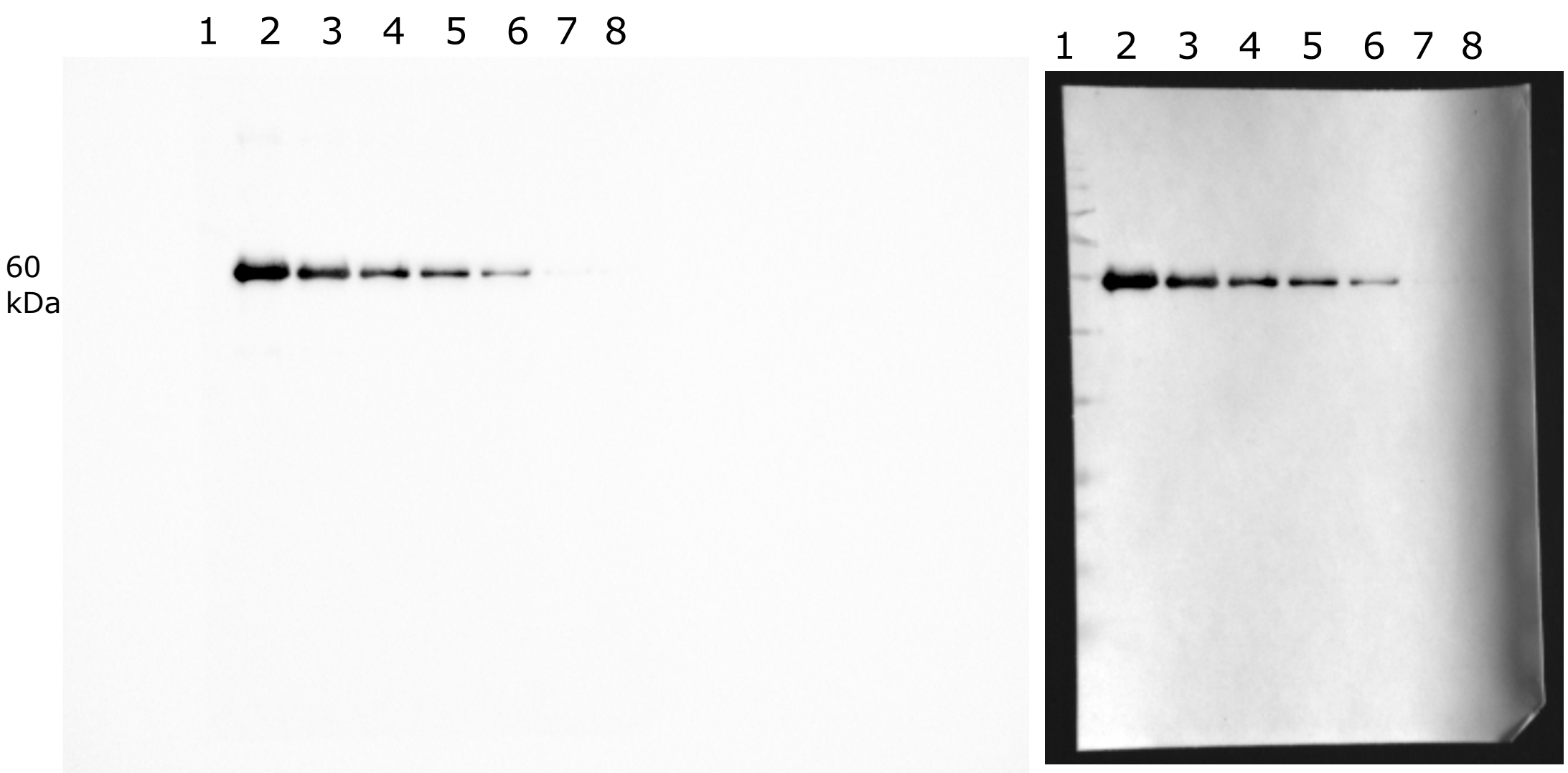

Raw image file for Fig 2B **(G8-5)**
Left blot: chemiluminescent image
Right: colorimetric merge

Samples
1 Molecular weight marker
2 250 ng BFT
3 125 ng BFT
4 62.5 ng BFT
5 31.25 ng BFT
6 15.625 ng BFT
7 7.8125 ng BFT
8 3.90625 ng BFT

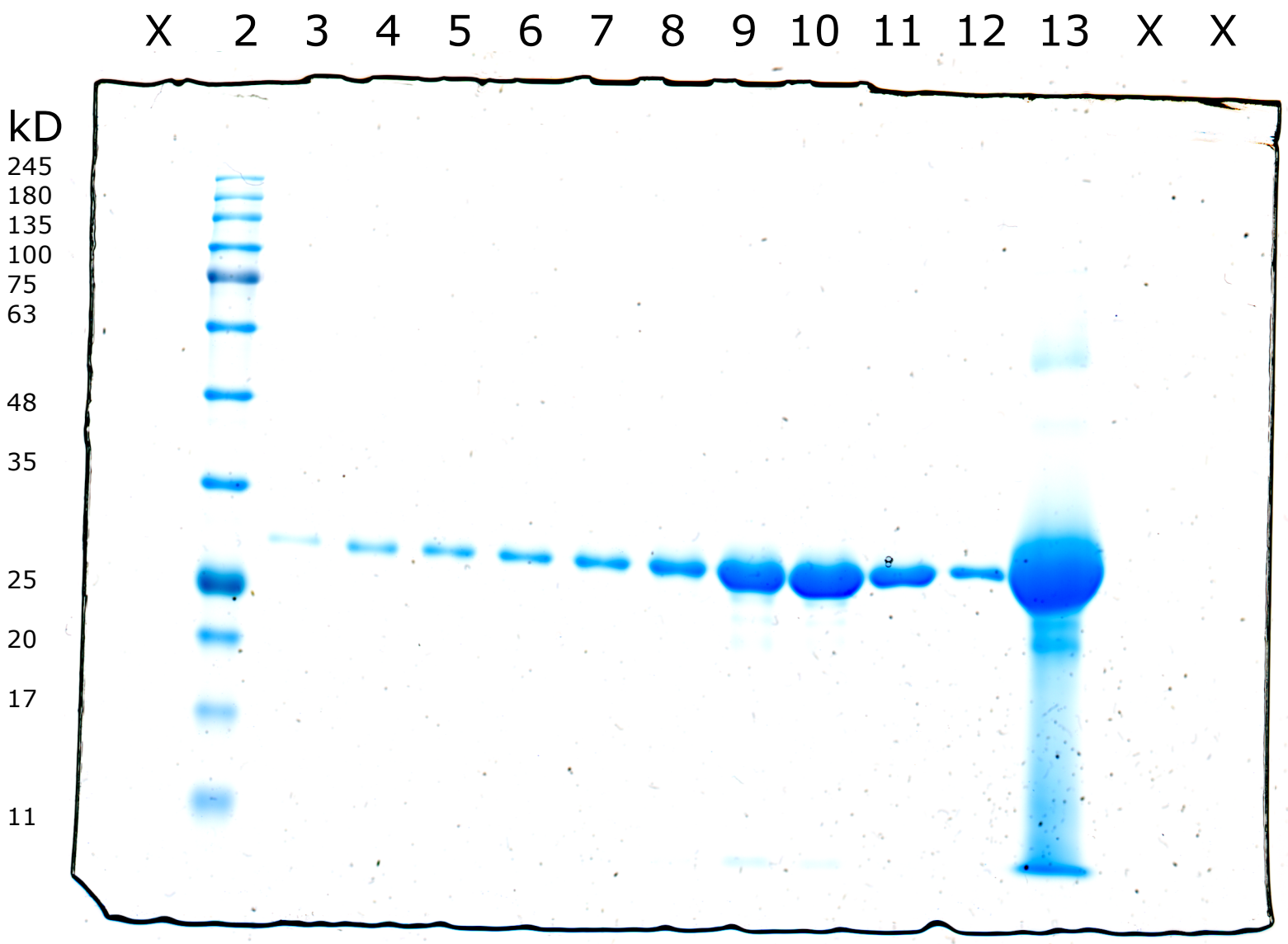

Raw image for Fig 1B (B8-1)
1 No sample
2 Molecular weight marker
3 B8-1 FPLC fraction 1
4 B8-1 FPLC fraction 2
5 B8-1 FPLC fraction 3
6 B8-1 FPLC fraction 4
7 B8-1 FPLC fraction 5
8 B8-1 FPLC fraction 6
9 B8-1 FPLC fraction 7
10 B8-1 FPLC fraction 8
11 B8-1 FPLC fraction 9
12 B8-1 FPLC fraction 10
13 B8-1 Magnetic bead eluate
14 No sample
15 No sample

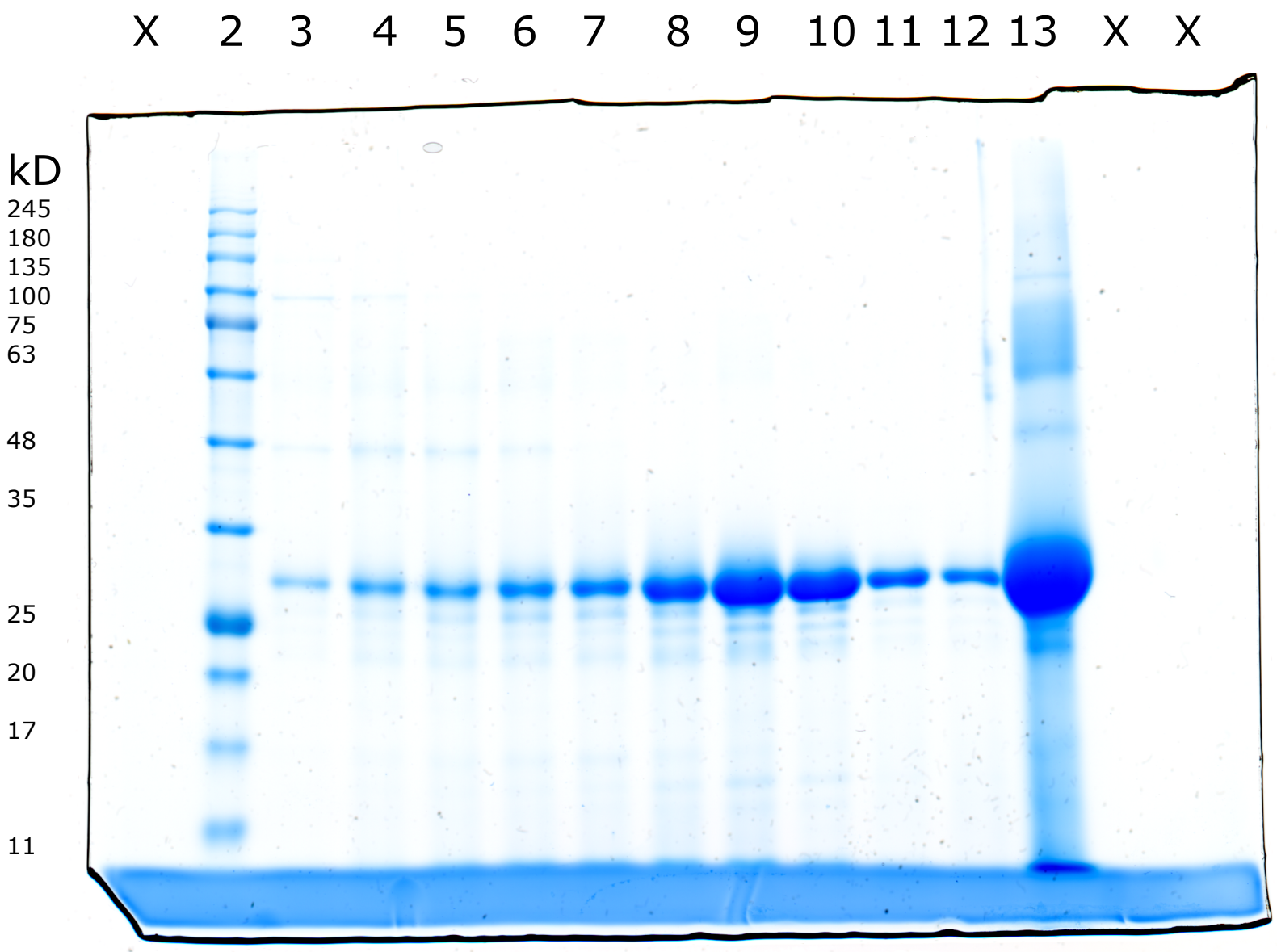

Raw image for Fig 1B (C2-6)
1 No sample
2 Molecular weight marker
3 C2-6 FPLC fraction 1
4 C2-6 FPLC fraction 2
5 C2-6 FPLC fraction 3
6 C2-6 FPLC fraction 4
7 C2-6 FPLC fraction 5
8 C2-6 FPLC fraction 6
9 C2-6 FPLC fraction 7
10 C2-6 FPLC fraction 8
11 C2-6 FPLC fraction 9
12 C2-6 FPLC fraction 10
13 C2-6 Magnetic bead eluate
14 No sample
15 No sample

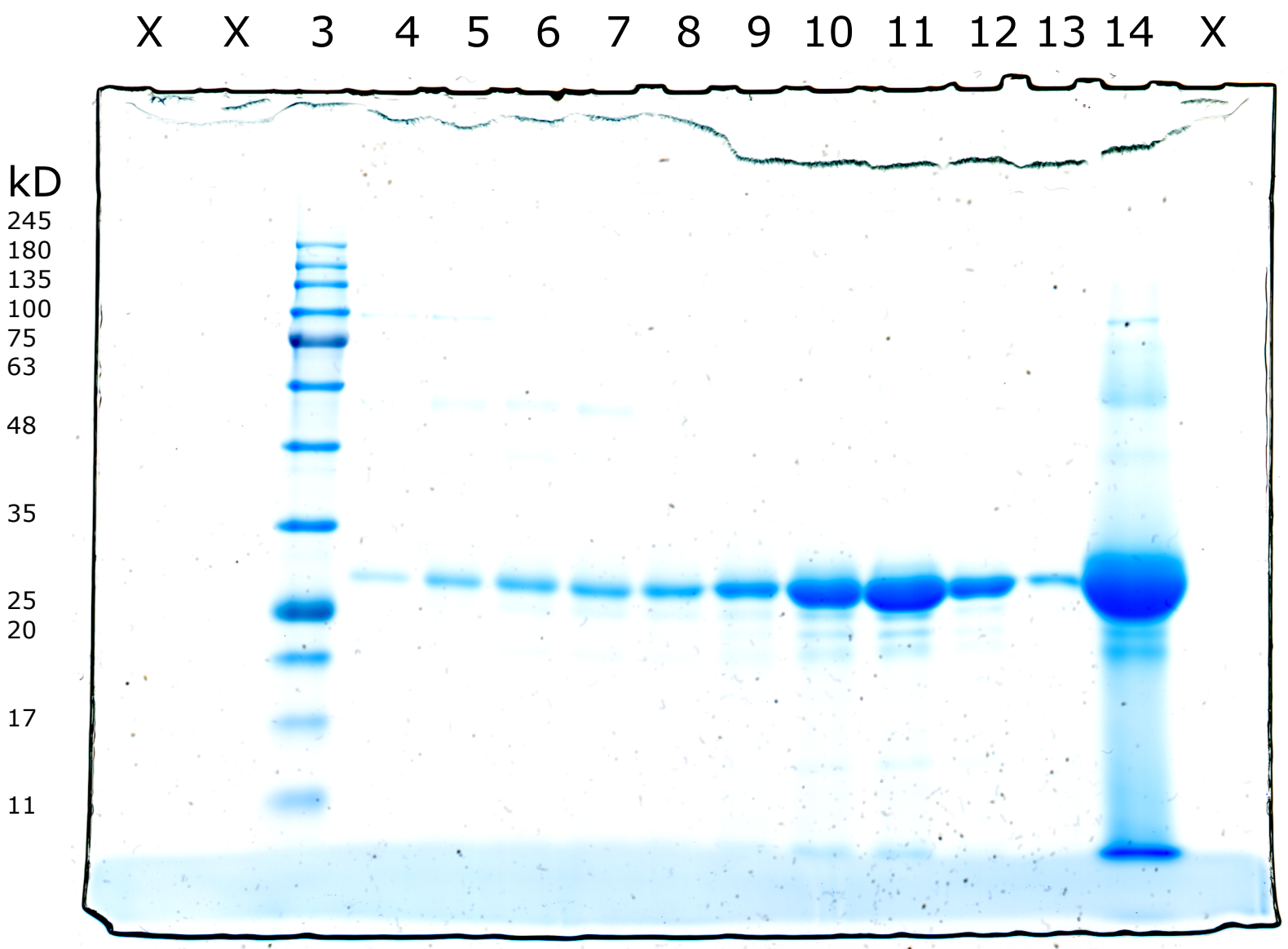

Raw image for Fig 1B (G8-5)
1 No sample
2 No sample
3 Molecular weight marker
4 G8-5 FPLC fraction 1
5 G8-5 FPLC fraction 2
6 G8-5 FPLC fraction 3
7 G8-5 FPLC fraction 4
8 G8-5 FPLC fraction 5
9 G8-5 FPLC fraction 6
10 G8-5 FPLC fraction 7
11 G8-5 FPLC fraction 8
12 G8-5 FPLC fraction 9
13 G8-5 FPLC fraction 10
14 G8-5 Magnetic bead eluate
15 No sample
